# Microbiota of Healthy Dental Pulp Under the Omics Loupe

**DOI:** 10.3390/ijms26073232

**Published:** 2025-03-31

**Authors:** Alan Bérard, Florian Mauffrey, Nadia Gaïa, Alexandre Perez, Chiraz Chaabane, Jacques Schrenzel, Julian Grégoire Leprince, Serge Bouillaguet, Vladimir Lazarevic

**Affiliations:** 1 Division of Cariology and Endodontology, University Clinics of Dental Medicine (CUMD), University of Geneva, 1211 Geneva, Switzerland; alan.berard@unige.ch (A.B.); julian.leprince@unige.ch (J.G.L.); 2 Genomic Research Laboratory, Division of Infectious Diseases, Department of Medicine, Geneva University Hospitals, University of Geneva, 1205 Geneva, Switzerland; mauffreyflorian@gmail.com (F.M.); nadia.gaia@genomic.ch (N.G.); chiraz.chaabane@genomic.ch (C.C.); jacques.schrenzel@genomic.ch (J.S.); vladimir.lazarevic@genomic.ch (V.L.); 3 Unit of Oral Surgery and Implantology, Division of Oral and Maxillofacial Surgery, Department of Surgery, Geneva University Hospitals, University of Geneva, 1205 Geneva, Switzerland; alexandre.perez@unige.ch; 4 Bacteriology Laboratory, Division of Laboratory Medicine, Department of Diagnostics, Geneva University Hospitals, 1205 Geneva, Switzerland

**Keywords:** microbiota, metataxonomics, 16S ribosomal RNA, next-generation sequencing, qPCR quantification, spike-in controls, dental pulp

## Abstract

This study aims to contribute to the understanding of the dental pulp microbiome through metataxonomic analysis of pulp tissues from clinically healthy impacted third molars in 17 patients. Samples from coronal pulp, outer radicular dentin, and negative controls were included. Each sample was spiked with a known bacterial standard. Sequencing of the 16S rRNA V3–V4 region revealed similarity in bacterial taxonomic profiles. The bacterial DNA was detected in all pulp samples, primarily originating from reagent contaminants. Although some oral taxa appeared more abundant in pulp than in dentin or controls, contaminant species overwhelmingly dominated. Most of the extracted DNA was of human origin, rather than bacterial. Our findings question the concept of a core microbiome in healthy pulp and highlight the critical need for rigorous controls in pulpal microbiome studies.

## 1. Introduction

Maintaining the vitality and function of caries-affected dental pulps through procedures referred to as “vital pulp therapies” has received renewed interest over the last decade, driven by a desire to develop minimally invasive treatment solutions [1]. These approaches are recommended in the recent S3-level guidelines from the European Society of Endodontology [1] and rely on the concept that the remaining pulp tissue exhibits minimal or no inflammation, retains the potential to produce calcified tissues, and is free of bacteria. The significance of achieving a bacteria-free status of the dental pulp has been clearly emphasized as a key prerequisite for the success of such procedures [1]. However, defining what constitutes bacteria-free pulp tissue is not straightforward.

Sound teeth are usually regarded as free from resident bacteria because the enamel and cementum are thought to act as impermeable barriers to bacterial penetration. However, a recent metataxonomic study based on next-generation sequencing (NGS) of the bacterial 16S rRNA gene has challenged this concept by detecting bacterial DNA in the pulp tissue of pristine, healthy teeth [2]. This finding has raised concerns, as some of the bacteria uncovered are not typically associated with the oral microbiome [3,4,5,6] but were common among different subjects.

The precision of bacterial detection using NGS approaches may be compromised by various factors, including challenges related to sampling, study design and data analysis. In the case of the oral cavity, a rigorous sampling procedure is essential to prevent contamination from bacteria near the collection site. Techniques such as rubber dam isolation, tooth disinfection, access preparation with sterile burs and DNA-free paper points for bacterial DNA collection have been recommended to minimize the risk of contamination. Additionally, contaminating DNA may be introduced through exposure to the laboratory environment or may originate from PCR reagents and DNA extraction kits [7,8,9].

These technical limitations can affect the accuracy of the metataxonomic and metagenomic analyses, particularly when assessing the relative abundance of taxa in low-biomass material communities. Therefore, it is crucial to include appropriate negative controls to correct for potential external contamination and positive controls to ensure that the NGS process functions as intended [6,10]. The use of spike-in bacteria has been proposed to ensure both qualitative and quantitative control throughout the entire metataxonomic or metagenomic workflow, from the initial disruption of bacterial cells to bioinformatic analysis [11,12,13].

While much of the existing literature has focused on caries-associated microbiota and the risk factors influencing lesion development and progression, the microbial status of healthy pulp tissue remains poorly understood. Establishing a reliable baseline of the pulp microbiome is a crucial first step toward understanding the microbial dynamics involved in pulp infections and the early stages of caries-related lesions. To further explore the dental pulp microbiome, we performed a 16S rRNA gene-based metataxonomic analysis of clinically healthy impacted teeth. For a more robust assessment, we incorporated negative controls and spike-in calibrators.

## 2. Results

### 2.1. DNA Load in Pulp Samples

**Figure 1:**
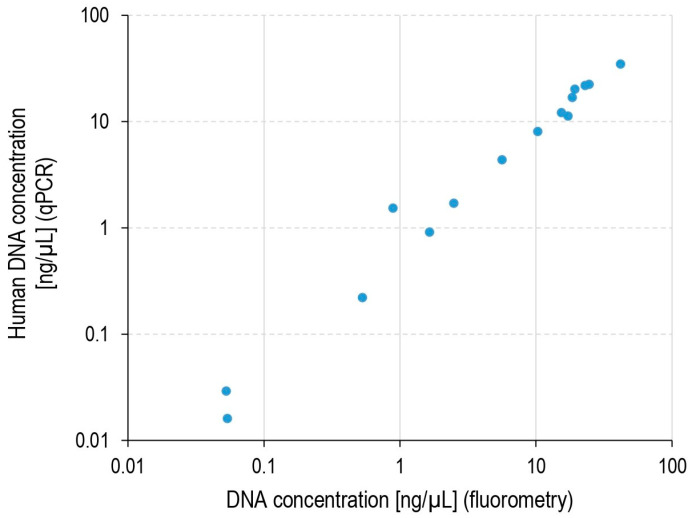
DNA concentration in pulp extracts estimated by qPCR and fluorometric assays. The qPCR test targeted the human beta-actin gene. For samples #3 and #4, no material was available to perform qPCR (fluorometric estimations were 18 and 13.7 ng/µL, respectively).

qPCR quantification was performed to estimate the human cell load in the extracted pulp. DNA concentrations obtained by qPCR targeting the human beta-actin gene and the fluorometric quantification of the overall DNA extracted were highly concordant (Figure 1). This result suggests that human DNA represents the bulk of the total extracted DNA. The estimated median human cell load per extracted pulp was 1.6 × 10^5^ (range 3.2 × 10^2^–6.9 × 10^5^). The differences in estimated human DNA were due to variable amounts of pulp tissue recovered from the teeth and possibly due to the loss of some DNA during DNA extraction and purification.

### 2.2. Bacteria in Dental Pulp, Root Dentin and Negative Controls

**Figure 2:**
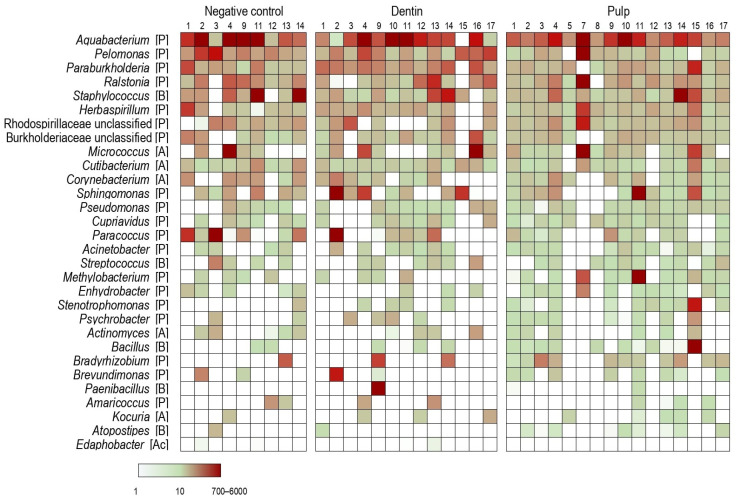
Dominant genera found in the three sample types. The top 25 genera for each sample were selected based on median abundance, and in cases of zero median, by mean abundance. Abundance is shown in cell equivalents, according to the color scale at the bottom. Bacterial phyla corresponding to each genus are represented as: [A], Actinomycetota; [Ac], Acidobacteriota; [B], Bacillota; [P], Pseudomonadota.

Seventeen dental pulp samples and their corresponding controls spiked with a known number of calibrator bacterial cells enabled the rough quantification of bacterial species through a metataxonomic analysis approach. Samples in which one or both spiked calibrators remained undetected were removed from the analysis. Additionally, two samples were removed due to an unexpectedly high ratio between the number of reads of *Allobacillus halotolerans* to *Imtechella halotolerans* spikes (435 and 2603, respectively) as these values contrasted those of other samples, which ranged between 0.5 and 3.1. The remaining pulp (n = 16), dentin (n = 13), and reagent control (n = 9) samples were further analyzed. The bacterial profiles of the three sample types were substantially similar (Figure 2). Among the top eight genera with the highest overall median relative abundance, six belonged to the order Burkholderiales.

**Figure 3:**
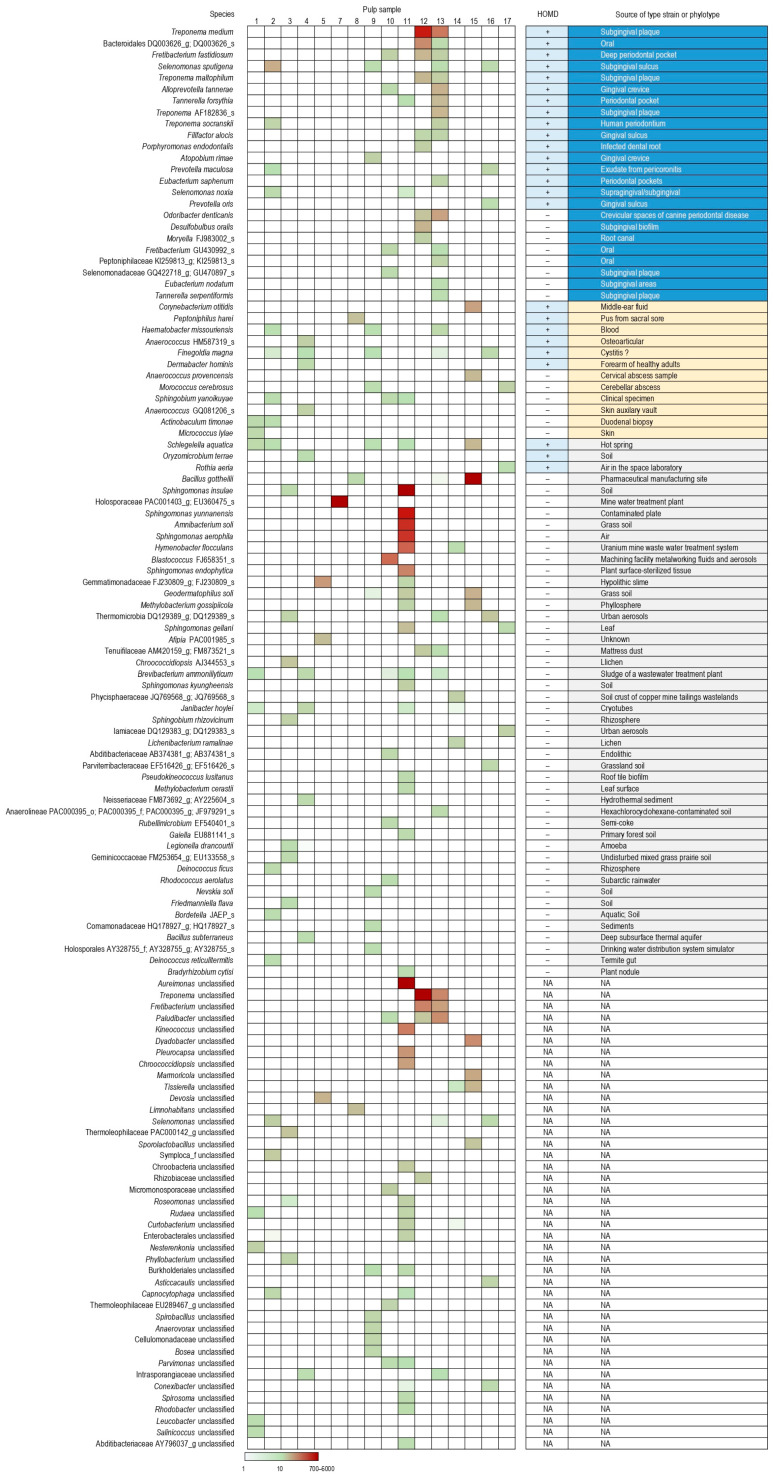
Bacterial species with sporadically and substantially higher abundance in pulp vs. dentin and negative controls. Presented are the species with ≥20 cell equivalents in at least one pulp sample and a ≥20-fold higher estimated abundance in at least one pulp sample relative to the highest abundance among all dentin and negative control samples. Only estimated abundances of >1 cell equivalent are indicated by the color scale. HOMD, Human Oral Microbiome Database; “+/–”, species present/absent on the HOMD list; NA, not applicable as these taxa represent summed-up zOTUs that remained unclassified at the species level.; “_g” and “_s” denote genus- and species-level phylotypes (taxa with names not yet validated), respectively. Cells highlighted in blue, yellow, and gray correspond to species-type strains or phylotypes recovered from the oral cavity, other body sites, or the environment, respectively.

Several species and genera were significantly more abundant in pulps compared to corresponding dentin or negative control samples (Table 1). However, following the correction for multiple comparisons, none of these differences remained statistically significant. Despite this lack of significance, it is important to consider that the hypothetical pulp microbiota may differ among individuals. Hence, pulp-associated bacterial species may not appear as significantly increased in statistical comparison with dentin or control samples. Therefore, an additional search was performed for species with a substantially higher abundance in at least one pulp sample than in all dentin and negative control samples. A total of 124 species were identified with ≥20 cell equivalents in at least one pulp sample and at least a 20-fold lower estimated abundance in all dentin and negative control samples (Figure 3). Twenty-four of these species are known oral bacteria, and an additional eight are also related to the oral microbiome, although not listed in the Human Oral Microbiome Database (HOMD) [14]. Among “non-oral” species, 6 correspond to bacteria initially isolated from different body sites, and 44 are from the environment. It is noteworthy that in only two samples (#12 and #13), an oral species or genus—specifically *Treponema medium* or the *Treponema* genus—had a higher relative abundance than any obvious contaminant within the same sample.

## 3. Discussion

For several decades, the pulp tissues of sound teeth have been considered a sterile environment. Recent metataxonomic data have challenged this assumption by reporting the existence of an apparent core pulpal microbiome [2]. In our study, using a similar approach, we identified DNA in all pulp samples, and in all dentin and negative controls. The most abundant bacterial genera identified in dental pulp overlapped with those of dentin and negative controls, indicating likely reagent contamination. This was further supported by the fact that six of the top eight detected genera with the highest overall median relative abundance belonged to the order Burkholderiales, the species of which are common contaminants in microbiome analyses [15]. Furthemore, the genera *Ralstonia*, *Acinetobacter*, *Staphylococcus*, *Micrococcus* and *Corynebacterium*, previously described as dominant or core members of a healthy pulp microbiome [2], were found to be likely contaminants, given their similar abundance and high prevalence across the pulp and control samples.

Our study identified several bacterial species that were sporadically present in pulp samples while being absent or present at a substantially lower abundance in control samples. The majority of these species correspond to known oral anaerobes and environmental bacteria, with a small subset of bacteria typically found in non-oral body sites. Among the genera and species “enriched” in dental pulp (Table 1) compared to the dentin and control samples, *Treponema, Fretibacterium*, *Finegoldia magna* and *Fusobacterium nucleatum* were recognized oral taxa. *Schlegelella aquatica* and members of the genera *Microbacterium* and *Atopostipes* were considered both oral and environmental microorganisms [16,17,18]. *Corynebacterium* KI515728_s, initially isolated from nares, was listed in the HOMD (*Corynebacterium accolens*), while *Corynebacterium afermentans*, originally isolated from the blood, is also a common blood cultures contaminant [19]. Other species and genera found to be more abundant in the pulp were typically environmental. However, it is important to note that, except for two cases (*Treponema*), the oral species and genera “enriched” in the pulp samples were scarce, and were less abundant than reagent contaminant organisms.

Very recently, the absence of culturable bacteria in healthy dental pulps has been reaffirmed by Arruda-Vasconcelos et al., who showed that vital healthy pulps were negative for viable bacteria, as well as endotoxins and inflammatory mediators [20]. However, it is conceivable that bacteria may be transiently present in healthy dental pulps at low abundance, which is insufficient to trigger the development of pulp disease. Possible sources of these bacteria include transient bacteremia [2,21] and “translocation” from undetected lesions in the surrounding tissues. These bacteria may not necessarily remain viable over time, but their DNA could be detectable over a longer period before being cleared. Additionally, despite decontamination efforts, the possibility of contamination by oral bacteria/DNA from the tooth surface during pulp extraction cannot be entirely ruled out.

Metataxonomic and metagenomic studies typically estimate the relative abundance of microbial species, which may lead to misleading conclusions since the relationship between relative and absolute abundance is unpredictable [22]. In the present work, the use of spike-in bacteria provided insights into the absolute abundance of bacterial DNA in the pulp microbiome. Spike-in calibrators allowed the whole process to be controlled, from DNA extraction to data analysis. Thus, samples with undetected spike-ins or distorted ratios of spiked organisms were excluded, as their observed microbial profiles and abundances would likely not properly reflect the actual ones. While spike-in based quantification offers advantages, some challenges relative to the extremely low bacterial DNA burden in the pulp still remain.

Our study is limited by its reliance on DNA-based detection, which cannot distinguish between viable bacteria and residual DNA. Additionally, we did not assess functional or immune responses to determine whether the detected microorganisms trigger a host reaction. Future research can apply complementary approaches, such as RNA-based analyses to assess bacterial activity and immune marker profiling from both blood and saliva to evaluate immune responses. Integrating microbial profiling with host immune markers and metabolic signals will clarify the physiological state of microbial presence and its pathological relevance in pulp tissues.

Building on our work and the prevailing view that healthy pulp lacks culturable bacteria, the application of the concept of pulpal eubiosis and dysbiosis remains challenging. This concept is more applicable to microbiomes with higher microbial loads, such as those in the intestinal, vaginal, or specific oral niches. However, it remains possible that the persistence of some bacteria in the pulp could represent a pathway toward disease. Such a shift might indicate early, subtle immune dysregulation and serve as a preclinical marker of future pathology. Identifying the threshold between a silent bacterial presence and a potentially threatening state is therefore a critical challenge.

## 4. Materials and Methods

### 4.1. Sample Collection and Processing

This study included 17 patients (18–30 years old), scheduled for the surgical removal of their impacted mandibular third molars, for preventive reasons. All patients were in good general health, asymptomatic and did not receive any prophylactic antibiotics prior to surgical extraction. According to the Swiss Federal Act on Research involving Human Beings (https://www.fedlex.admin.ch/eli/cc/2013/617/en, accessed 12 May 2022), the use of anonymized extracted teeth is considered to fall outside of the scope of this legislation, so the need for local ethics committee approval was waived. Tests were performed on non-erupted teeth to minimize the contact of the tested sample with the oral microbiome that could potentially contaminate the virgin pulp by a crack in the enamel if the tooth erupted.

Prior to extraction, all patients rinsed their mouths for 1 min with 0.2% chlorhexidine mouthwash (Plak-Out liquid, KerrHawe SA, Bioggio, Switzerland) and the oral mucosa at the surgical extraction site was scrubbed with 10% povidone iodine for 1 min (Betadine, Mundipharma Medical Company, Basel, Switzerland). After local anesthesia (Ubistesin forte, 3M ESPE Dental Products, Rüschlikon, Switzerland), a full-thickness mucoperiosteal flap was elevated and osteotomy was performed using a carbide sterile bur mounted on a surgical handpiece (CHIROPRO 3rd Gen, Bien-Air Dental SA, Bienne, Switzerland), cooled with saline solution irrigation. During osteotomy, care was taken to reduce the risk of surgical trauma to the tooth as much as possible.

The extracted teeth were processed under a laminar flow hood and carefully controlled under the operating microscope (Zeiss Extaro 300, Oberkochen, Germany) to ensure that the surgical procedure had not created routes for pulpal contamination, such as cracks, exposed dentinal tubules or iatrogenic fractures [23]. The outer surface of the tooth was wiped off with 1% sodium hypochlorite, rinsed with 100 µL of ZymoBIOMICS DNase/RNase-free water (Zymo Research, Irvine, CA, USA) and placed into a disposable Petri dish to collect dentin chips from the root surface (dentin samples) after removing any remnants of the dental follicle. A periodontal scaler was used to collect dentin chips that served as a control for potential contamination from the dental surface, procedural materials and reagents.

The teeth were then transferred into a new Petri dish and a mesio-distal groove was cut in the occlusal enamel to allow for the placement of a scalpel blade (10, KLS Martin Group, Tuttlingen, Germany) that received a brief load to force the tooth to fracture. This procedure allowed pulp tissues to be exposed minimizing the risk of external contamination that might occur during the drilling of an access cavity with rotative burs. From each patient, one specimen of coronal pulp (pulp samples) was collected in 100 µL of ZymoBIOMICS water.

To control for material and reagent DNA contamination, 100 µL of ZymoBIOMICS water was recovered by rinsing an unused scalpel blade over a Petri dish (negative controls). Collected pulp, dentin and control samples were immediately frozen and stored at −80 °C until further processing.

### 4.2. DNA Extraction and Quantification

Samples (100 µL each) were transferred to Microbial DNA Free 2 mL tubes with 2.8 mm ceramic beads (Omni International, Kennesaw, GA, USA) and 750 µL of ZymoBIOMICS Lysis solution [from the ZymoBIOMICS DNA Miniprep kit (Zymo Research)]. After the addition of 10 µL of the ZymoBIOMICS spike-in Control I (High Microbial Load) (Zymo Research) diluted in DNA/RNA Shield (Zymo Research), containing 500 cells of *Imtechella halotolerans* and *Allobacillus halotolerans*, the mixture was shaken at maximum speed for 15 min on a Vortex-Genie 2 with a horizontal tube holder (Scientific Industries, Bohemia, NY, USA). Following a brief spin, a 650 µL aliquot of the supernatant was transferred to a ZR BashingBead Lysis Tube (0.1 & 0.5 mm) from the ZymoBIOMICS DNA Miniprep Kit. The volume was adjusted to 750 µL with ZymoBIOMICS Lysis Solution and bead beating was performed for 15 min on a Vortex-Genie 2 with a horizontal tube holder. From this point, the ZymoBIOMICS DNA Miniprep protocol was followed according to the manufacturer’s instructions. DNA was eluted in 50 µL water and stored at −20 °C. Purified DNA was quantified using the Qubit fluorometer 2.0 with Qubit dsDNA BR and HS Assay Kits (Thermo Fisher Scientific, Carlsbad, CA, USA) as recommended by the manufacturer.

### 4.3. qPCR Assay

The concentrations of human DNA were determined by qPCR, targeting the human beta-actin reference gene as described previously, using 1 µL of a non-diluted DNA extract [24].

### 4.4. Amplicon Sequencing

Genomic DNA was dissolved in 1 µL of Tris (0.1 M, pH 8) and sent to LGC Genomics (Berlin, Germany) for DNA amplification and sequencing. Bacterial 16S rRNA gene sequences were amplified using a nested PCR approach as previously described [25]. Briefly, for the first PCR, the V3–V6 region of the bacterial 16S rRNA genes was amplified using 6 µL of the DNA extract obtained from clinical samples or negative controls with primers 314F (5′-CCTACGGGAGGCAGCAG-3′) and 1061R (5′-CRRCACGAGCTGACGAC-3′). For the second round of the nested-PCR, 1 µL of the PCR product from the first PCR was used to amplify the V4–V5 region of the bacterial 16S rRNA genes. The nested-PCR was carried out using the barcoded primers 515F-Y (5′-GTGYCAGCMGCCGCGGTAA-3′) and 962R-jed (5′-CCGYCAATTYMTTTRAGTTT-3′). The first and the second PCRs were each carried with an initial pre-denaturation at 96 °C for 2 min, followed by 20 cycles using the following parameters: 96 °C for 15 s, 50 °C for 30 s, and 70 °C for 90 s. Following the use of Agencourt AMPure XP beads (Beckman Coulter, Brea, CA, USA) and MinElute columns (Qiagen, Venlo, The Netherlands) for amplicon purification, Illumina libraries were constructed using the Ovation Rapid DR Multiplex System 1–96 (NuGEN Technologies, Leek, The Netherlands) and sequenced 2 × 300 on an Illumina MiSeq instrument using the MiSeq Reagent Kit v3 (Illumina, San Diego, CA, USA).

### 4.5. Bioinformatics Analysis

Paired reads were quality filtered and joined using PEAR v.0.9.11 (-m 400 -n 350 -v 25 -p 0.0001 -u 0) [26]. Merged sequence reads were clustered into zero-radius operational taxonomic units (zOTUs) using UNOISE3 [27] from USEARCH v.11.0.667 [28], which resolves differences of as little as one nucleotide and provides taxonomic resolution superior to conventional 97% OTUs. This pipeline also removes putative chimeric sequences. From the sample dataset we removed zOTUs matching any of the following criteria: (1) <90% identity presented to reference EzBioCloud 16S database sequences (downloaded on 3 February 2020) as revealed by USEARCH (-id 0.90 -query_cov 0.99); (2) representations of ≤10 counts. zOTUs were classified using the EzBioCloud 16S database [29] via MOTHUR’s v.1.48.0 command classify.seqs (method=wang cutoff=80) [30]. Sequencing data were submitted to the European Nucleotide Archive (ENA) under accession number PRJEB50897 with the following codes: A for control, B for dentin and C for pulp samples.

### 4.6. Assessment of the Bacterial Load

To roughly determine the load of the bacterial species in each sample, zOTU counts belonging to the same species or the same genus were summed up. The estimation of the absolute abundance of a given taxon was based on the ratio between its read counts and those of the two calibrators—*I*. *halotolerans* (*Ih*; 3 copies of the 16S rRNA gene per genome) and *A*. *halotolerans* (*Ah*; 7 copies of the 16S rRNA gene per genome)—each spiked at 500 cells. The absolute abundance (*AB*) of taxon *_X_* was calculated as follows: *AB_X_* = (*N_X_*/*N_Ih_* × 500 × 3/*Copy16S_X_* + *N_X_*/*N_Ah_* × 500 × 7/*Copy16S_X_*)/2, where *N* is the number of reads assigned to a given taxon and *Copy16S_X_* is the median number of 16S rRNA copies per genome of species *X* [31]. If *Copy16S* was not available for a given species, the value of the corresponding higher ranked taxon was used instead.

## 5. Conclusions

Although the present results confirm the presence of bacterial DNA in all specimens of healthy pulp, they do not seem to support the concept of a “core microbiome“ in healthy pulp. Because of the low bacterial DNA burden in the pulp tissue, multiple negative controls and DNA quantification are of the utmost importance for the assessment of the pulpal microbiome.

## Figures and Tables

**Table 1:** Species and genera that were differentially abundant between the dental pulp and dentin samples. The Wilcoxon signed-rank test was used to test the significance of changes between the matched pulp and dentin or negative control samples.

Taxon		Abundance [Median (Minimum; Q1; Q3; Maximum)]			*p* *	
		Negative Control	Dentin	Pulp	Pulp vs. Negative Control	Pulp vs. Dentin
**Species**	*Atopostipes* unclassified	0 (0;0;0;0.12)	0 (0;0;0;0.36)	2.1 (0;0;6.5;15)	**0.022**	**0.009**
	*Bacillus gottheilii*	0 (0;0;0;0)	0 (0;0;0;0)	0.014 (0;0;0.04;5995)	**0.022**	**0.006**
	*Brevibacterium ammoniilyticum*	0 (0;0;0;0)	0 (0;0;0;0)	0 (0;0;5.9;32)	0.059	**0.036**
	*Brevundimonas albigilva*	0 (0;0;0.13;295)	0 (0;0;0.035;0.61)	0 (0;0;17;65)	0.402	**0.035**
	*Corynebacterium afermentans*	0 (0;0;0;215)	0 (0;0;0;0)	0 (0;0;1.7;119)	0.402	**0.036**
	*Cutibacterium acnes*	60 (19;38;123;267)	58.4 (24;45;101;154)	30 (0;16;54;216)	**0.008**	**0.027**
	*Edaphobacter* unclassified	0 (0;0;0.65;3)	0.50 (0;0;0.83;4.2)	0 (0;0;0;0.77)	0.100	**0.042**
	*Finegoldia magna*	0 (0;0;0;0.085)	0 (0;0;0;0)	0 (0;0;5.4;26)	0.093	**0.022**
	*Fretibacterium* unclassified	0 (0;0;0;0)	0 (0;0;0;0)	0 (0;0;0.039;304)	**0.036**	**0.022**
	*Fusobacterium nucleatum*	0 (0;0;0;36)	0 (0;0;0;0)	0 (0;0;8.7;36)	0.295	**0.036**
	*Corynebacterium* KI515728_s	0 (0;0;0;22)	0 (0;0;0;0.018)	0 (0;0;8.1;18)	0.402	**0.022**
	*Microbacterium* unclassified	0 (0;0;0;46)	0 (0;0;0;0.035)	0 (0;0;16;36)	0.106	**0.022**
	*Paraburkholderia* unclassified	150 (35;101;215;473)	296 (0;171;345;446)	132 (0;93;173;593)	0.359	**0.040**
	*Paracoccus aestuarii*	2.5 (0;0.23;183;1321)	0 (0;0;0.19;2.2)	0 (0;0;12;27)	0.076	**0.032**
	*Pelomonas* unclassified	230 (152;198;307;663)	226 (50;122;385;507)	71 (0;36;144;1656)	**0.004**	**0.006**
	*Pseudomonas poae*	0 (0;0;0.14;51)	0 (0;0;0.093;17)	11 (0;1.8;21;50)	0.098	**0.003**
	*Rothia amarae*	0 (0;0;0;111)	0 (0;0;0;30)	0 (0;0;3.5;157)	0.100	**0.035**
	*Schlegelella aquatica*	0 (0;0;0;0)	0 (0;0;0;0.21)	0.030 (0;0;11;136)	**0.036**	**0.014**
**Genus**	*Treponema*	0 (0;0;0;0)	0 (0;0;0;0)	0 (0;0;0.12;1482)	**0.036**	**0.036**
	*Bacillus*	0 (0;0;0.36;21)	0.039 (0;0;1.6;36)	6.3 (0;0.058;20;5995)	**0.039**	0.126
	*Cutibacterium*	60 (19;57;123;268)	73 (24;45;121;193)	37 (0;22;79;339)	**0.020**	0.244
	*Edaphobacter*	0 (0;0;0.65;3)	0.50 (0;0;0.83;4.2)	0 (0;0;0;0.77)	0.100	**0.042**
	*Finegoldia*	0 (0;0;0;0.085)	0 (0;0;0;0)	0 (0;0;5.4;26)	0.093	**0.022**
	*Fretibacterium*	0 (0;0;0;0)	0 (0;0;0;0)	0.01 (0;0;0.068;447)	**0.036**	**0.014**
	*Fusobacterium*	0 (0;0;0;36)	0 (0;0;0;0)	0 (0;0;8.7;36)	0.295	**0.036**
	*Microbacterium*	0 (0;0;0;46)	0 (0;0;0;0.035)	0 (0;0;16;36)	0.106	**0.022**
	*Paraburkholderia*	150 (35;101;215;473)	296 (0;171;345;446)	132 (0;93;173;593)	0.359	**0.040**
	*Pelomonas*	230 (152;198;307;663)	226 (50;122;385;507)	71 (0;36;144;1656)	**0.004**	**0.006**
	*Pseudoalteromonas*	0 (0;0;0.011;23)	0 (0;0;0;0.0044)	0 (0;0;0.012;11)	0.554	**0.036**
	*Schlegelella*	0 (0;0;0;0)	0 (0;0;0;0.21)	0.030 (0;0;11;136)	**0.036**	**0.014**

* *p* values < 0.05 before Benjamini–Hochberg correction for multiple testing are indicated in bold. Q1 first quartile; Q3, third quartile.

## Data Availability

Sequencing data were submitted to the European Nucleotide Archive (ENA; www.ebi.ac.uk/ena) under study number PRJEB50897.
